# Cyclophosphamide responsive primary angiitis of the CNS in a 61-year-old female 

**DOI:** 10.5414/NP300513

**Published:** 2012-09-25

**Authors:** Jay Rosenberg, Ali Mahta, Kavya Koppula, Ewa Borys, Santosh Kesari

**Affiliations:** 1 Department of Neurology, Scripps Memorial Hospital,; 2 Department of Neurosciences Moores UCSD Cancer Center, UC San Diego, and; 3 Department of Pathology, Neuropathology Division, UC San Diego, La Jolla, CA, USA

## Letter to the Editor

**Figure 1:**
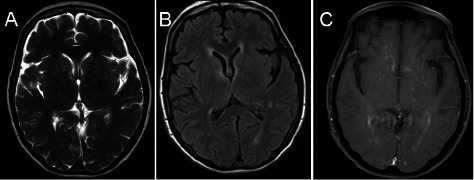
Figure 1. Axial Brain MRI sequences prior to treatment. A: T2 weighted image, B: FLAIR showing abnormal signal prolongation in periventricular areas, C: post contrast T1 image showing mild diffuse enhancement.

Dear Sir – A 61-year-old female with no significant past medical history presented with flu like symptoms including fever, chills and headaches after returning from a trip to Hawaii. First saw her primary care physician who advised conservative management. A couple of days later, she presented with worsening of her headaches and was admitted for meningitis work up. The neurologic exam was unremarkable. A lumbar puncture revealed a pleocytosis with 206 WBC and an elevated total protein of 75 mg/dl. PCR was negative for West Nile, HSV and enterovirus. Malaria smear, leptospira, and cryptococcal antigen were also negative. A brain MRI was unremarkable except some slight periventricular T2 changes near the anterior horn of the lateral ventricle. She was eventually discharged with presumptive diagnosis of aseptic meningitis. However, she failed to improve with persistent nausea and unsteadiness of gait and vision gradually became blurry. A repeat brain MRI showed multifocal abnormal T2 prolongation in periventricular regions (Figure 1A, B) with contrast enhancement on T1-weighted images (Figure 1C). In addition, MRI of cervical, thoracic and lumbar spine showed significant T2 signal changes in the cervical and thoracic cord with enhancement, both patchy and confluent. She underwent a brain biopsy and the preliminary pathology was concerning for lymphoma or vasculitis. Intravenous methylprednisolone was initiated afterwards and the patient improved immediately. 

**Figure 2:**
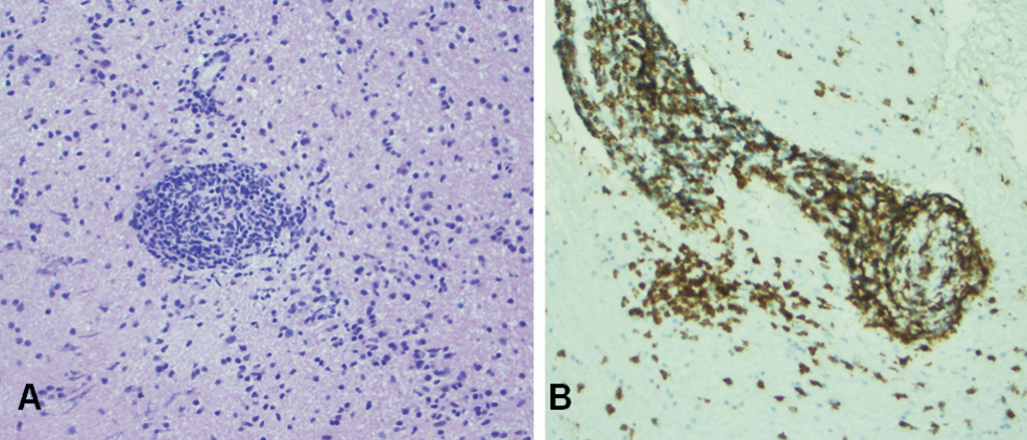
Figure 2. A: Obliterated vascular lumen by reactive endothelial cells and infiltration of mature lymphocytes. B: Immunohistochemical staining for CD3 showing infiltration of T-lymphocytes.

The pathology revealed white matter with perivascular cellular infiltrates. Lumens were nearly obliterated by reactive endothelial cells and, percolating through the blood vessel walls, were populations of predominantly small mature appearing lymphocytes (Figure 2A) accompanied by reactive microglia or macrophages. The inflammatory infiltrates showed no significant cytologic atypia. The blood vessel walls, while perturbed by the passing lymphocytes and microglia, did not show fibrinoid necrosis. No granulomas were identified. Surrounding parenchyma was hypercellular as a result of infiltration by scattered T-lymphocytes and reactive microglia accompanied by some fibrous gliosis. The immunohistochemical staining for CD3 revealed that the angiocentric infiltrates were predominantly T-lymphocytes (Figure 2B). Only rare CD20 positive B lymphocytes were present. These changes are most consistent with lymphocytic vasculitis. 

**Figure 3:**
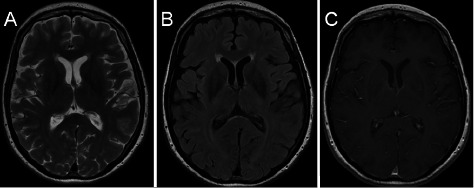
Axial Brain MRI sequences after treatment. A: T2 weighted image, B: FLAIR, C: post contrast T1 image showing resolution of previously noted lesions.

She was started on pulse intravenous cyclophosphamide (cytoxan). At 1 year follow-up, she is feeling quite well, almost completely back to her normal self. A follow-up brain MRI demonstrated a significant resolution of the previously noted lesions (Figure 3). 

Vasculitides, in general, are very broad spectrum and can affect any organs including brain either isolated or in the context of a systemic process such as lupus erythematosus or polyarteritis nodosa (PAN). However, primary CNS vasculitis or primary angitis of central nervous system (PACNS) is a rare disease characterized by inflammation and necrosis of walls of medium and small sized vessels in the CNS only [1, 2]. The clinical manifestations can be heterogeneous including headache, stroke, myelopathy and encephalopathy. Imaging and other para-clinical findings are nonspecific. Cerebrospinal fluid (CSF) pleocytosis and protein elevation are commonly seen. However, pathology is the gold standard diagnostic test. Spinal cord involvement is rarely seen in PACNS and most of the time is associated with brain lesions [3, 4]. Goertz et al. [4] reported a patient with an isolated homogeneously enhancing lesion in the spinal cord which turned out to be PACNS [4]. The pattern of spinal cord involvement in our case was multifocal with both confluent and patchy enhancement which makes it different from previously reported cases. 

Current treatment recommendations are steroid therapy with intravenous pulse cyclophosphamide (cytoxan). Other alternative treatment options include: rituximab, azathioprine, methotrexate and mycophenolate mophetil [1, 2, 3, 4, 5, 6]. The number of cases of PACNS reported in the literature thus far is about 700 [2]. Given its multifarious clinical presentations, PACNS usually mimics other pathologies such as tumors. In our case, the initial impression was aseptic meningitis but later on the imaging findings and the frozen section report raised the suspicion of lymphoma. However the permanent pathology ascertained the diagnosis of CNS vasculitis and as there was no evidence of other organ involvement, PACNS is our final diagnosis. The patient initially responded to steroids quickly; however her gait and lower limb ataxia gradually worsened over a period of 3 weeks. At that time a pulse cyclophosphamide regimen was recommended. Pulse cyclophosphamide therapy, in addition to steroids, has been used for treatment of PACNS, however it is important to rule infectious causes of vasculitis before starting any immunosuppressive treatment [2, 7]. In our case, the extensive work up did not reveal any evidence of infectious etiologies. The relapse rate in PACNS is estimated to be around 25%, so it is important to keep in mind that a regular follow up is essential for a proper care in PACNS patients [2]. We conclude that PACNS is a relatively rare pathology that is a great masquerader and creates diagnostic and therapeutic challenges. Pulse cyclophosphamide therapy has been recommended and should be considered early as a therapeutic option. 

## Acknowledgment 

This work was supported in part by grants from NIH 3P30CA023100-25S8 to S. Kesari.
